# Automated semi-quantitative amyloid PET analysis technique without MR images for Alzheimer’s disease

**DOI:** 10.1007/s12149-022-01769-x

**Published:** 2022-07-11

**Authors:** Etsuko Imabayashi, Naoyuki Tamamura, Yuzuho Yamaguchi, Yuto Kamitaka, Muneyuki Sakata, Kenji Ishii

**Affiliations:** 1 Research Team for Neuroimaging, Tokyo Metropolitan Geriatric Hospital and Institute of Gerontology, 35-2 Sakae-cho, Itabashi-ku, Tokyo, 173-0015 Japan; 2 Department of Molecular Imaging and Theranostics, Institute for Quantum Medical Science, Quantum Life and Medical Science Directorate, National Institutes for Quantum Science and Technology (QST), 4-9-1 Anagawa, Inage, Chiba 263-8555 Japan; 3 Nihon Medi-Physics Co., Ltd., 3-4-10 Shinsuna, Koto-ku, Tokyo, 136-0075 Japan

**Keywords:** Alzheimer’s disease, Automated Aβ-PET analysis, Centiloid, ^18^F-flutemetamol-PET, Semi-quantitative Aβ analysis

## Abstract

**Objective:**

Although beta-amyloid (Aβ) positron emission tomography (PET) images are interpreted visually as positive or negative, approximately 10% are judged as equivocal in Alzheimer’s disease. Therefore, we aimed to develop an automated semi-quantitative analysis technique using ^18^F-flutemetamol PET images without anatomical images.

**Methods:**

Overall, 136 cases of patients administered ^18^F-flutemetamol were enrolled. Of 136 cases, five PET images each with the highest and lowest values of standardized uptake value ratio (SUVr) of cerebral cortex-to-pons were used to create positive and negative templates. Using these templates, PET images of the remaining 126 cases were standardized, and SUVr images were produced with the pons as a reference region. The mean of SUVr values in the volume of interest delineated on the cerebral cortex was compared to those in the CortexID Suite (GE Healthcare). Furthermore, centiloid (CL) values were calculated for the 126 cases using data from the Centiloid Project (http://www.gaain.org/centiloid-project) and both templates. ^18^F-flutemetamol-PET was interpreted visually as positive/negative based on Aβ deposition in the cortex. However, the criterion "equivocal" was added for cases with focal or mild Aβ accumulation that were difficult to categorize. Optimal cutoff values of SUVr and CL maximizing sensitivity and specificity for Aβ detection were determined by receiver operating characteristic (ROC) analysis using the visual evaluation as a standard of truth.

**Results:**

SUVr calculated by our method and CortexID were highly correlated (*R*^2^ = 0.9657). The 126 PET images comprised 84 negative and 42 positive cases of Aβ deposition by visual evaluation, of which 11 and 10 were classified as equivocal, respectively. ROC analyses determined the optimal cutoff values, sensitivity, and specificity for SUVr as 0.544, 89.3%, and 92.9%, respectively, and for CL as 12.400, 94.0%, and 92.9%, respectively. Both semi-quantitative analyses showed that 12 and 9 of the 21 equivocal cases were negative and positive, respectively, under the optimal cutoff values.

**Conclusions:**

This semi-quantitative analysis technique using ^18^F-flutemetamol-PET calculated SUVr and CL automatically without anatomical images. Moreover, it objectively and homogeneously interpreted positive or negative Aβ burden in the brain as a supplemental tool for the visual reading of equivocal cases in routine clinical practice.

**Supplementary Information:**

The online version contains supplementary material available at 10.1007/s12149-022-01769-x.

## Introduction

As of 2021, three amyloid positron-emission tomography (PET) tracers, ^18^F-florbetapir, ^18^F-florbetaben, and ^18^F-flutemetamol, are approved as pharmaceutical diagnostic agents by the Ministry of Health, Labour, and Welfare in Japan. These tracers are indicated for PET imaging of the brain to estimate β-amyloid (Aβ) neuritic plaque density in adult patients with cognitive impairment being evaluated for Alzheimer's disease (AD) and other causes of cognitive decline. Readers visually interpret the PET images distinctly as defined for each tracer, but not quantitatively, and classify them as positive or negative depending on the Aβ deposition in the brain. Regarding the positivity/negativity of Aβ deposition in the brain, the inter-rater agreement of visual assessment of ^11^C-Pittuberg compound B (PiB) [1–3] PET images, the most commonly used amyloid PET tracer worldwide, was higher (κ = 0.9) than those of ^18^F-florbetapir [4, 5], ^18^F-florbetaben [6], and ^18^F-flutemetamol [7 − 9] (κ = 0.8 each). Approximately 10% of Aβ-PET images are interpreted as equivocal as a border case between positivity and negativity, causing false-positive/negative results [10 − 12]. There have been reports that a semi-quantitative analysis using cortical standardized uptake value ratio (SUVr) to the cerebellar cortex or pons was useful for the evaluation of equivocal cases [3, 10, 11]. However, the SUVr is influenced by and varies according to differences in tracers, PET scanners, and/or imaging protocol such as radioactivity, time before images are acquired, and scan time, and hence, it is not suitable for quantification analysis of amyloid imaging [13]. This causes issues in a multicenter study.

One of the methods to solve this problem is the centiloid (CL) scale developed by Klunk et al. [13]. Briefly, CL values are calculated by converting the SUVr of each ^18^F-amyloid PET image to the SUVr obtained from images at 50–70 min postinjection of ^11^C-PiB and standardizing the semi-quantitative amyloid imaging measures to a scale from 0 to 100. The 0-anchor is intended to represent a definitively non-amyloid brain, while the 100-anchor is intended to represent the amount of global amyloid deposition found in a typical mild-to-moderate AD patient [13]. The CL methodology allows the calculation of a calibrated measure of Aβ binding for different tracers at different institutes if data are acquired with a consistent acquisition/reconstruction methodology [13]; therefore, this method has been adopted in recent clinical trials [13–16]. Furthermore, it has been reported that the CL method is useful for the positive/negative evaluation of Aβ burden in equivocal cases by visual interpretation [17].

Generally, MR images are required for anatomical standardization of PET images, followed by the calculation of SUVr using the standardized PET images; then, the resulting SUVr is converted to the CL values by referring to the recommended protocol of the Global Alzheimer’s Association Information Network (http://www.gaain.org). Therefore, it is difficult to utilize the CL method for PET images without the corresponding MR images, in clinical practice. To overcome this, several groups have reported quantitative analysis methods to calculate CL values using only PET images without anatomical imaging data such as MRI or CT images [18–20]. We also developed a new technique that enabled to standardize PET images anatomically without MR images, which can set the composite volume of interest (VOI) and calculate SUVr and CL values in the VOIs automatically with simple operations. In addition, we found an optimal cutoff value for each SUVr and CL value in the composite VOI for separating positive and negative Aβ deposition in the brain by the receiver operating characteristic (ROC) curve, based on the visual evaluation of ^18^F-flutemetamol-PET as a standard of truth (SOT). Our semi-quantitative analysis technique is versatile and may improve the objectivity and reproducibility of the evaluation with ^18^F-flutemetamol-PET for AD as a supplemental tool in routine clinical practice.

## Materials and methods

### Participants

Data of 136 participants (40 men and 96 women) with a mean age of 79.2 years (range 68–86 years) were obtained from Tokyo Metropolitan Geriatric Hospital and Institute of Gerontology (TMIG) between July 2020 and January 2021. The selection criteria included patients diagnosed with AD, mild cognitive impairment (MCI), or cognitive unimpaired at our center; those who underwent both MRI (T1-weighted imaging) and PET scan using ^18^F-flutemetamol within the past 6 months; and those who were analyzed with VSRAD™ (Eisai Co., Ltd., Tokyo, Japan) (but not a requirement). The exclusion criteria were patients with poor quality MR and PET images (*e.g.,* low resolution, distortion, artifacts, and lack of whole-brain coverage); patients with brain tumors or intracerebral hemorrhage that would interfere with the analysis; and patients deemed inappropriate by the principal investigator (K.I.). Among PET images from the 136 participants, images from 10 participants were used for creating positive and negative templates. The remaining 126 participants were classified as negative in 84 cases and positive in 42 cases for Aβ deposition by visual reading, and 11 of the negative and 10 of positive cases were classified as equivocal. The final clinical diagnosis was normal in 92 cases for normal, while in 34 cases, the diagnosis was MCI due to AD or no dementia due to AD. The other epidemiological characteristics of the participants were summarized in Table 1. The study was approved by the institutional ethics committee of TMIG, and written informed consent was obtained from all participants.

### MR and PET imaging

Amyloid PET images were obtained using an integrated PET/CT scanner, Discovery 710, and Discovery MI (GE Healthcare, Milwaukee, United States). At approximately 90 min postinjection of 180.9 ± 7.9 (range, 140.1–200.4) MBq ^18^F-flutemetamol synthesized by FASTlab™ (GE Healthcare), 30-min list-mode PET scan was started, and the PET images were reconstructed using the 3D ordered subset expectation maximization with a time-of-flight procedure (Iteration 4, subset 16, and filter cut off 4 mm).

MRI was performed using Achieva 1.5 T (Philips Healthcare, Andover, MA, the United States). T1-weighted 3D turbo field echo SENSE1 sequence was used for volumetric MRI. The clinical report including physical examination, MRI, and other clinical examinations were all obtained not earlier than 5 months before the PET/CT scan.

### Visual reading

PET/CT images were visually assessed by two experienced nuclear medicine specialists (E.I. and K.I.) certified by the Japanese Society of Nuclear Medicine and evaluated with the MRI results. The brightness of the pons on the PET image was adjusted to 90% of the maximum intensity of the color scale, and the accumulation of Aβ was evaluated as negative or positive in five regions of the brain (frontal lobe, temporal lobe, parietal lobe, posterior cingulate gyrus and precuneus, and striatum). If any one of the five regions was positive, it was judged as Aβ positive; if all five regions were negative, it was considered as Aβ negative. Participants ruled out as negative or positive because of mild accumulation and/or uncertain extent of Aβ in the brain were classified as equivocal by consensus between the two readers. The SUVr of the cerebral cortex-to-pons was measured using CortexID Suite (GE Healthcare) in all cases.

### Image processing

**Fig. 1:**
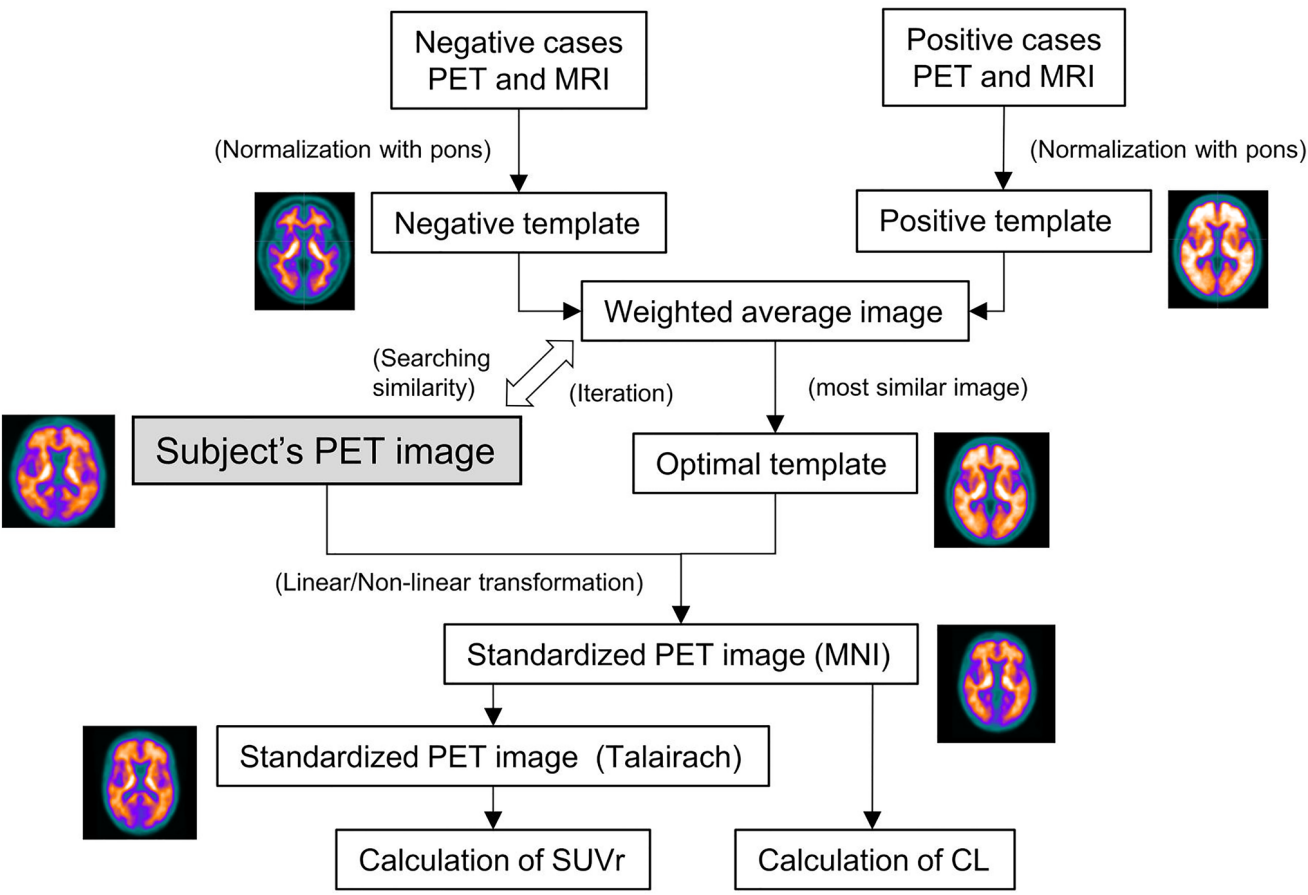
Workflow of the procedure to create the optimal templates for each subject and data analyses. CL: centiloid; SUVr: standardized uptake value ratio

Figure 1 shows the workflow of the procedure to create the optimal template for each participant and the data analyses. For the deformation of PET images, we used a program that implements a technique for representing deformation fields using basic functions as well as Statistical Parametric Mapping (SPM). This is an in-house program based on a previous report [21].

### PET template-based spatial normalization

Five deformed PET images each with the highest and lowest SUVr in cerebral cortex measured using the CortexID Suite were selected; arithmetic mean-based positive/negative templates were created. The accumulation of Aβ varied from patient to patient, and the standardization error increased with a single template. Therefore, the positive and negative templates were weighted from 0.1% to 99.9% and from 99.9% to 0.1% in 0.1% increments, and the weighted average was used as a candidate template for evaluating the similarity with the participant's PET images by Zero-mean normalized cross-correlation (ZNCC) [22], The candidate template with the highest similarity was adopted as the optimal template. The similarity was calculated as follows:

Step 1: Subject PET images were affine-transformed based on the averaged images of the positive and negative templates.

Step 2: ZNCC was calculated for the weighted-average template candidates in 0.1% increments and the affine-transformed images.

Step 3: The template candidate with the highest ZNCC value was adopted as the optimal template.

Finally, the PET images of 126 cases excluding the 10 cases used for the template creation were standardized directly based on the optimal template without MR images.

### Centiloid process

**Fig. 2:**
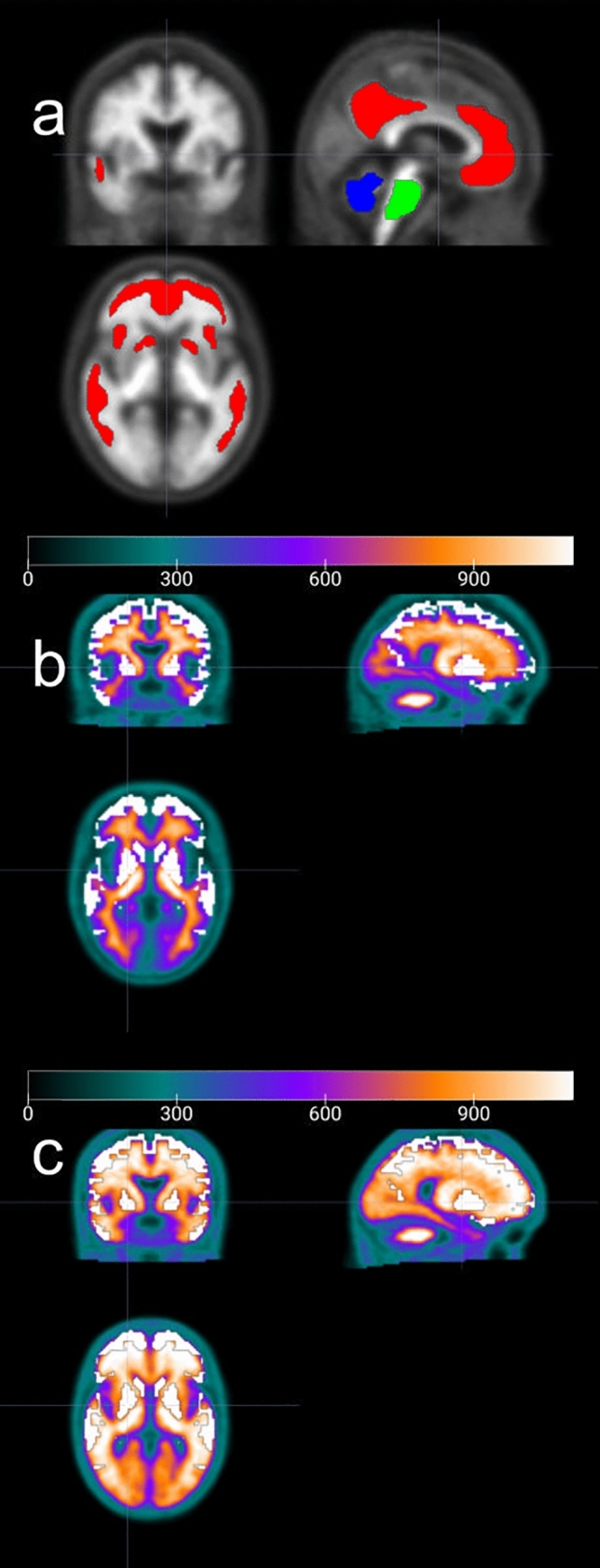
VOIs used for the calculation of semi-quantitative analyses (CL, SUVr). **a** The standard VOIs published in the Centiloid Project were used to calculate the CL values. The areas colored red, blue, and green show the VOIs put on the cerebral cortex, total cerebellum, and pons, respectively. The VOI on pons was used for the calculation of SUVr in each region of the brain. **b**, **c** For calculating SUVr, the VOIs (white) were drawn in the gray matter of frontal lobes, parietal lobes, temporal lobes, posterior cingulate gyrus, precuneus, and basal ganglia of negative **b** and positive **c** PET images, based on the Talairach Daemon. The VOIs of these six regions were integrated to calculate the Composite SUVr with the pons as the reference region. *CL* centiloid; *SUVr* standardized uptake value ratio

The SUVr values were converted to the CL values by referring to the method by Klunk et al. [13]. Firstly, the standard VOI of 1.0-mm pixel pitch published in the Centiloid Project (http://www.gaain.org/centiloid-project) was converted to 1.5-mm pixel pitch and used to calculate the CL values (Fig. 2a). The mean pixel values were measured in each VOI put on the cerebral cortex, total cerebellum, and pons; the SUVr in the cerebral cortex was calculated as the mean pixel value of the cerebral cortex divided by that of the total cerebellum; and then, the SUVr was transferred to CL values using the formula mentioned above (the mean pixel value of pons was used for the calculation of SUVr in each region of the brain). Secondly, the anatomical standardized images for the ^18^F-flutemetamol-PET images published in the Centiloid Project were deformed using arithmetic mean-based positive/negative templates created in this study, followed by calculation of SUVr_Flute_ for those images. Then, the SUVr values corresponding to the SUVr_PiB_ in the Centiloid Project were calculated from the SUVr_Flute_.

### Quantitative analyses

The anatomically standardized 126 PET images (Montreal Neurological Institute [MNI] coordinates) were transformed into Talairach standard brain PET images (Talairach) using the pre-determined deformation parameters from MNI standard brain to Talairach standard brain [23]. The MNI to Talairach deformation parameters were obtained by using the Talairach-shaped FDG-PET images included in NEUROSTAT (https://neurostat.neuro.utah.edu/) as templates and linearly and nonlinearly deforming the MNI-shaped PET images included in SPM. An in-house program based on previous reports [21] was used for this deformation.

The VOIs for each region (frontal lobes, parietal lobes, temporal lobes, posterior cingulate gyrus, precuneus, and basal ganglia) were drawn to measure the pixel values in only the gray matter to avoid counting signals from white matter, based on the Talairach Daemon (Fig. 2b, c), and the VOIs of these six regions were integrated to calculate the SUVr (defined as "Composite") with the pons as the reference region. To verify the validity of the automatically calculated Composite SUVr, the values were compared with those calculated by CortexID Suite, which is used worldwide for quantitative analysis of ^18^F-flutemetamol PET images. Furthermore, the CL values for each participant were calculated for the 126 anatomically standardized PET images (MNI) using the SUVr to CL value conversion formula mentioned above. Finally, the ROC curve was drawn using EZR [24] and the optimal CL cutoff using the evaluation of visual reading of ^18^F-flutemetamol-PET images as SOT was determined by ROC analysis, maximizing the Youden’s index (sensitivity + specificity—1). The area under the curve (AUC), sensitivity and specificity at the optimal CL value were then calculated.

### Statistics

The correlation between SUVr and CL values calculated by our method and SUVr calculated by CortexID Suite were examined with linear regression and coefficient of determination (R^2^) using Microsoft Excel 2016 (Microsoft Japan, Tokyo, Japan). The differences of SUVr and CL values calculated by the current method between the groups (negative, equivocal, positive) evaluated by visual reading were compared using the Mann–Whitney U test, and p < 0.05 was set as the level of significance. The reproducibility of evaluation between visual reading and semi-quantitative analysis for the participants was assessed using Cohen’s kappa.

## Results

### Validity of anatomical standardization

**Fig. 3:**
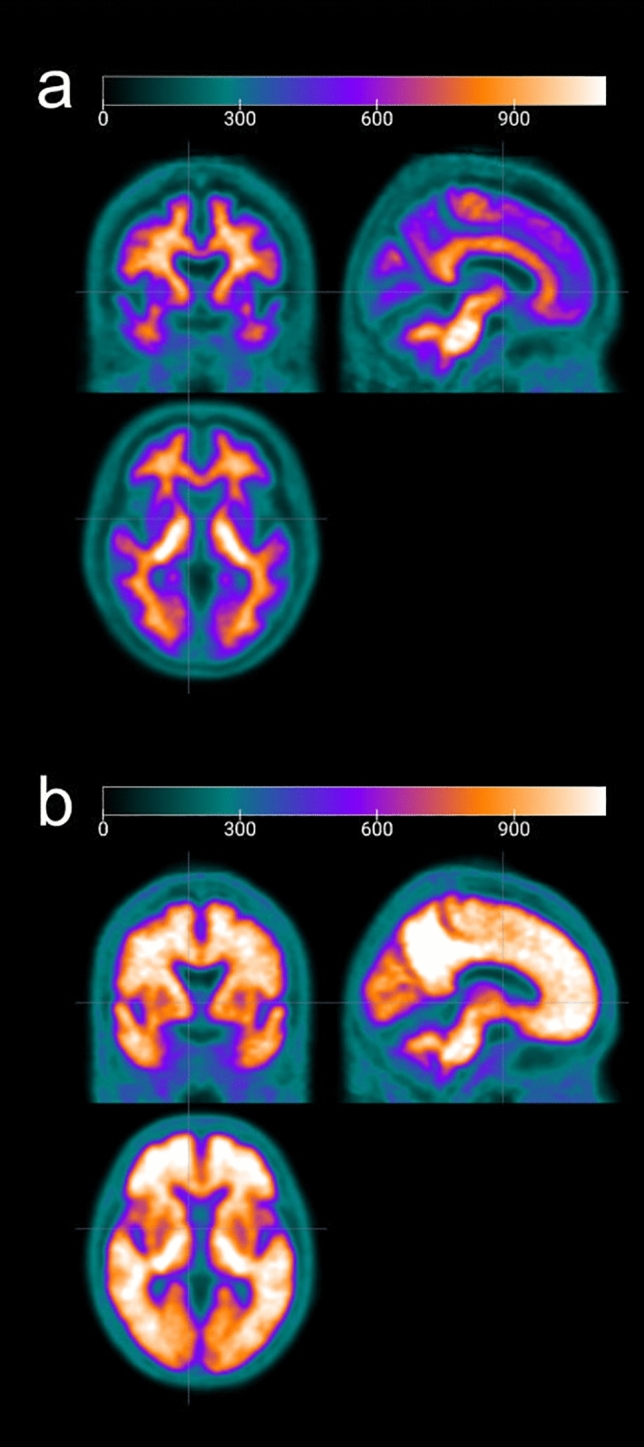
Arithmetic mean-based negative (a) and positive (b) standard brain templates. Each five PET images with the lowest and the highest value of SUVr in ^18^F-flutemetamol accumulation between cerebral cortex and pons were standardized anatomically by using DARTEL, then normalized based on the mean pixel in the pons. DARTEL: Diffeomorphic Anatomical Registration Through Exponentiated Lie Algebra; SUVr: standardized uptake value ratio.

**Fig. 4:**
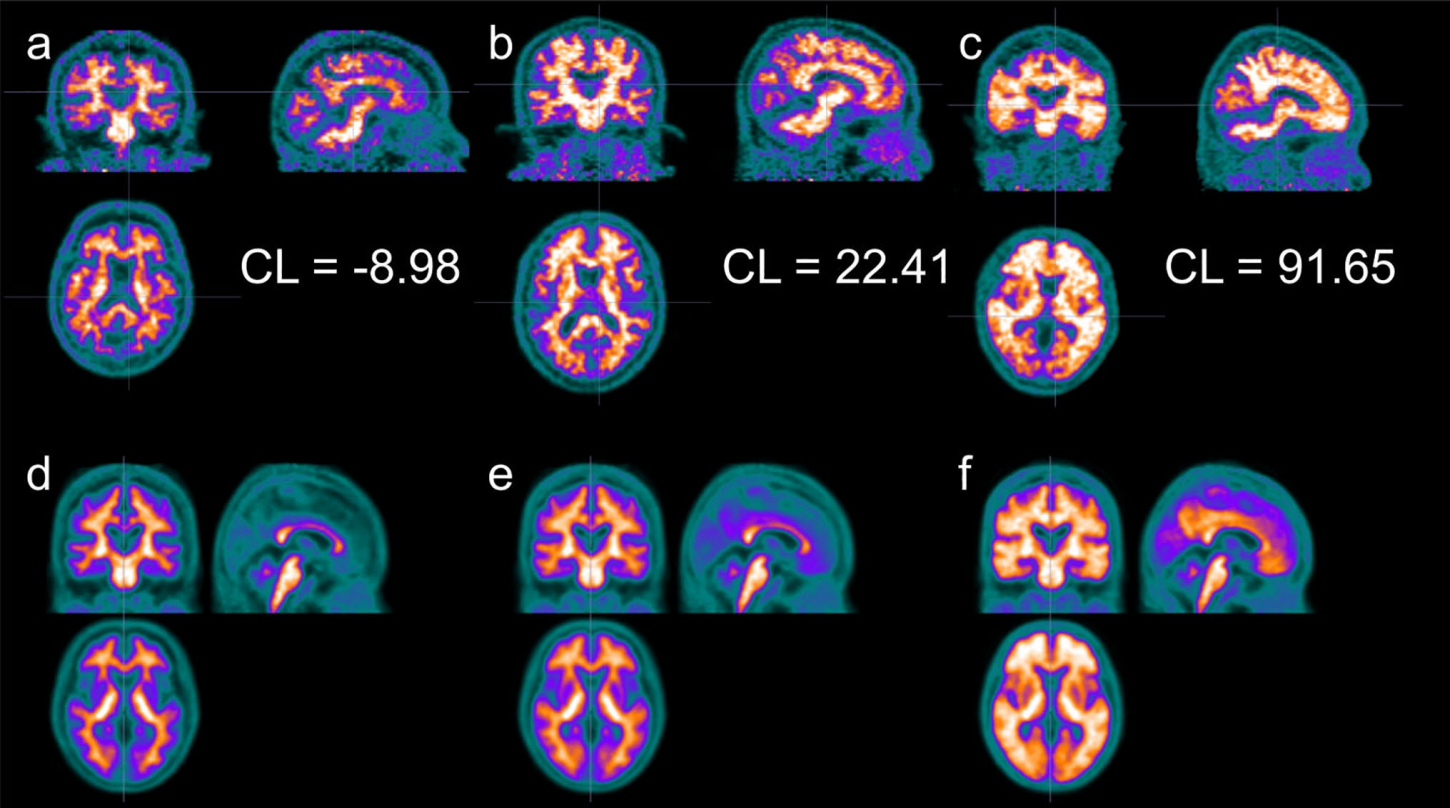
Examples of subject ^18^F-flutemetamol-PET images **a**−**c** and their optimal templates **d**−**f**. **a** and **d**, **b** and **e**, **c** and **f** are PET images and the optimal templates with the CL values of -8.98, 22.41, 91.65, respectively. *CL* centiloid.

Five PET images each with the lowest and highest value of SUVr in ^18^F-flutemetamol accumulation between the cerebral cortex and pons were standardized anatomically and then normalized based on the mean pixel in the pons. The negative and positive standard brain templates obtained by weighted averaging are shown in Fig. 3a, b, respectively. Then, the weighted average image of these templates was used as the optimal template to obtain the highest similarity to each participant PET image. For example, the optimal template (d − f) for each case (a − c) with CL values of -8.98 (a and d), 22.41 (b and e), and 91.65 (c and f) are shown in Fig. 4, respectively.

### Validity of SUVr

**Fig. 5:**
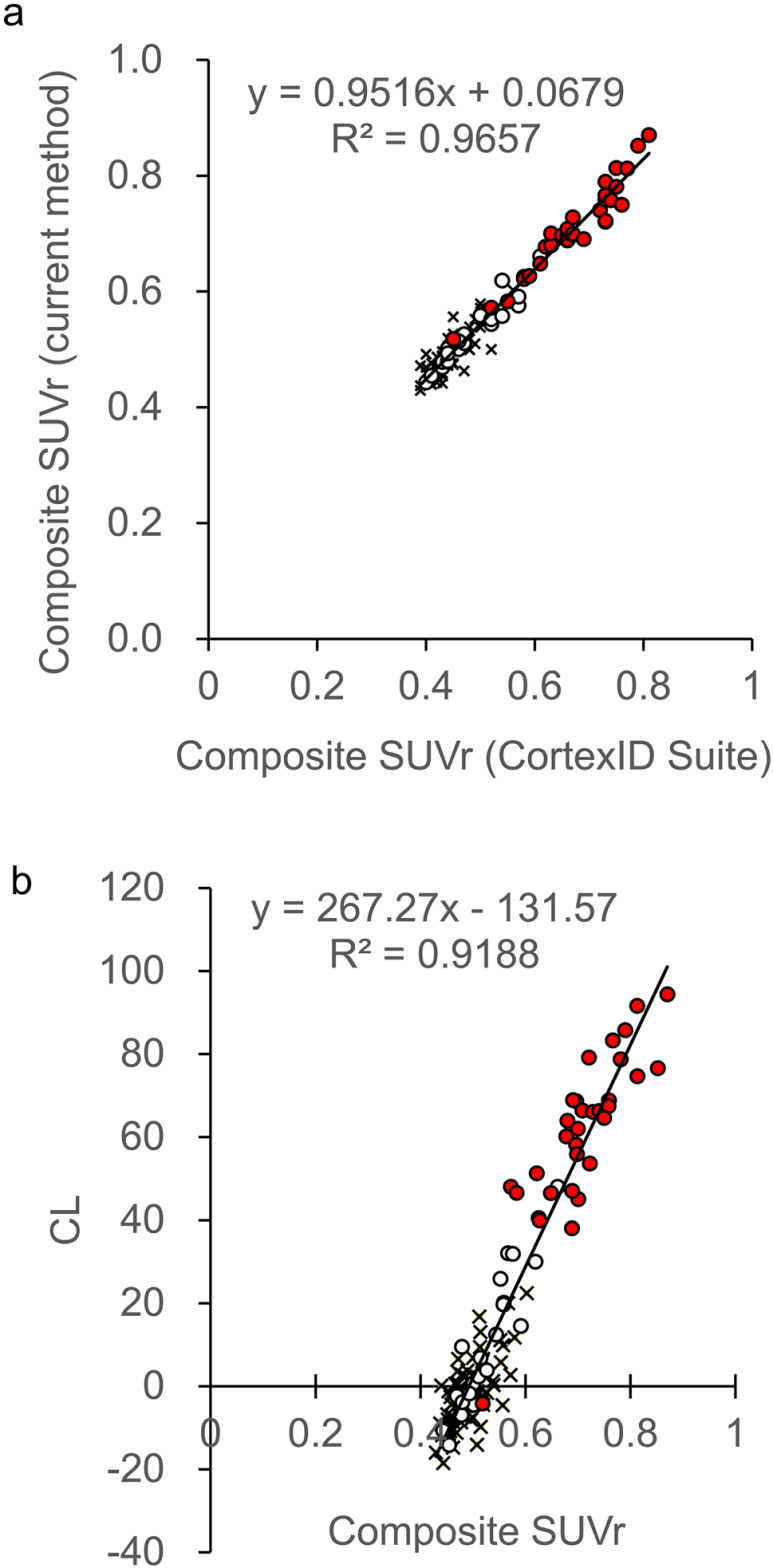
Dot plots between Composite SUVr calculated by the current method (Y-axis) and CortexID Suite (X-axis) (a) and between the CL values (Y-axis) and Composite SUVr (X-axis) calculated by the current method (b). Each dot shows negative (☓), equivocal (open circle), and positive (filled circle) cases evaluated by visual reading of ^18^F-flutemetamol PET imaging. CL: centiloid; SUVr: standardized uptake value ratio

To verify the validity of the composite SUVr calculated automatically using our semi-quantitative analysis method, we compared them with that calculated by CortexID Suite. As shown in Fig. 5A, the composite SUVr in the current method correlated well with that in Cortex ID Suite, with a coefficient of determination (R^2^) of 0.9657 (slope = 0.9516, intercept = 0.0679).

### Comparison with GAAIN data

The formula to convert the SUVr to the CL values directly was defined as follows: CL = 122.83 × SUVr_Flute_ − 126.13. The coefficient of determination (R^2^) between the CL value calculated by this conversion formula and the CL value published in the Centiloid Project was 0.983 (slope: 1.000, intercept: 0.538).

### Validity of the results of quantitative analyses

**Fig. 6:**
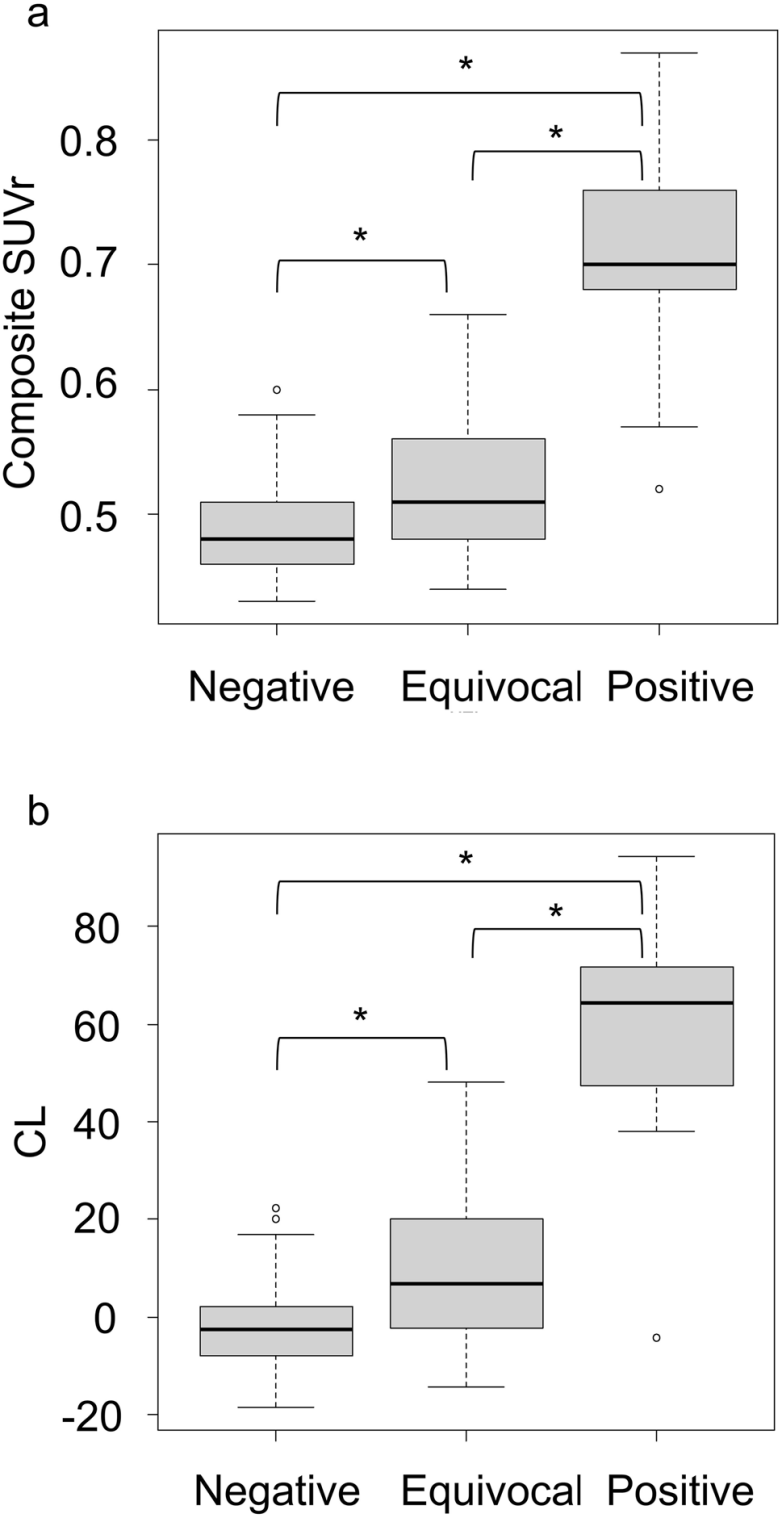
Visual reading against Composite SUVr and CL values. Box plots represent the distributions of Composite SUVr (a) and CL values (b) per negative, equivocal, and positive case assessed by the visual reading of ^18^F-flutemetamol-PET imaging. The box plot shows the median, first/third quartiles, and 1.5 times the inter-quartile range. * p < 0.01. CL: centiloid; SUVr: standardized uptake value ratio

The Composite SUVr and CL values of flutemetamol-PET imaging calculated by the current method showed a high correlation (R^2^ = 0.9188). The values of SUVr and CL assessed by visual reading are shown in Table 1 and Fig. 6. The mean ± standard deviation (median, range) of SUVr values in each negative, equivocal, and positive group classified by visual reading were 0.45 ± 0.04 (0.48, 0.39 − 0.55), 0.48 ± 0.05 (0.51, 0.41 − 0.61), and 0.65 ± 0.07 (0 0.70, 0.48 − 0.80), respectively. Similarly, those of CL values were − 1.91 ± 8.08 (− 2.50, − 18.5 − 22.4), 10.2 ± 16.1 (6.83, − 14.2 − 48.1), and 61.1 ± 18.9 (64.30, − 4.16 − 94.4), respectively. Significant differences in SUVr and CL values between all groups were observed (*p* < 0.01).

**Fig. 7:**
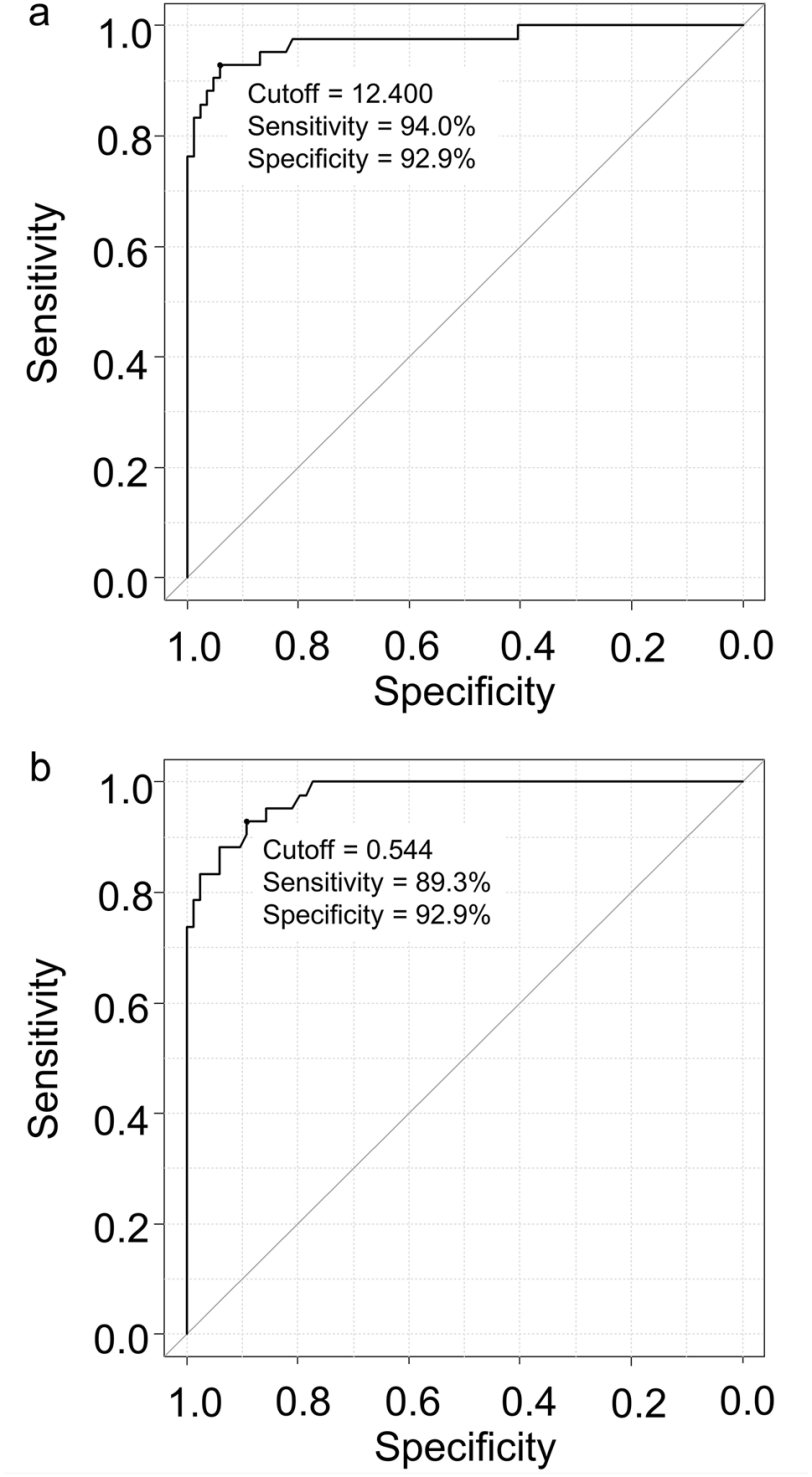
ROC curves for CL (a) and SUVr cutoff against the assessment of visual reading of ^18^F-flutemetamol-PET. Numbers in panels indicate the optimal cutoff value and the sensitivity and specificity at the cutoff value. CL: centiloid; ROC: receiver operating characteristic; SUVr: standardized uptake value ratio

We investigated the optimal cutoff value of CL units for separating positive and negative Aβ deposition in the brain by ROC curve analysis (Fig. 7), based on the evaluation of visual reading of ^18^F-flutemetamol-PET images as a SOT. As a result, the maximum value of AUC was 0.973 (95% CI: 0.943–1) and the optimal CL cutoff value was 12.400 for CL values, and the sensitivity and specificity at the optimal cutoff value were 94.0% and 92.9%, respectively (Fig. 7a). On the other hand, the maximum value of AUC, 95% CI, optimal cutoff, sensitivity, and specificity for SUVr were 0.977, 0.957 − 0.997, 0.544, 89.3%, and 92.9%, respectively (Fig. 7b). The reproducibility of the results of evaluation between visual reading and quantitative analysis at the optimal cutoff was examined using Cohen's kappa for all 126 cases (visual reading: 84 negative and 42 positive) and 21 equivocal cases (visual reading: 11 negative and 10 positive) (Table 2). In 126 participants, the optimal SUVr cutoff value was reassessed as 78 negative and 48 positive, and kappa coefficient was 0.7932, while those values for the optimal CL cutoff value were 82 negative and 44 positive, kappa coefficient was 0.8588. Both the quantitative analyses showed that 12 and 9 cases of the 21 equivocal cases were negative and positive, respectively, under the optimal cutoff values, and the kappa coefficient was 0.7123.

**Table 1:** Characteristics of patients

		Visual evaluation		
	All	Negative	Equivocal	Positive
Number	126	73	21	32
Age (range)	79.3 ± 3.9 (68 − 86)	78.6 ± 4.1 (68 − 86)	80.7 ± 3.6 (75 − 86)	80.1 ± 3.3 (72 − 86)
MMSE	27.7 ± 2.4 (21 − 30)	27.9 ± 2.3 (21 − 30)	28.1 ± 1.8 (24 − 30)	27.0 ± 2.7 (21 − 30)
Clinical diagnosis (normal, MCI)	90, 36	57, 16	16, 5	17, 15
SUVr	0.51 ± 01 (0.39 − 0.80)	0.45 ± 0.04 (0.39 − 0.55)	0.48 ± 0.05 (0.41 − 0.61)	0.65 ± 0.07 (0.48 − 0.80)
CL	16.1 ± 29.7 (-18.5 − 94.4)	-1.91 ± 8.08 (-18.5 − 22.4)	10.2 ± 16.1 (-14.2 − 48.1)	61.1 ± 18.9 (-4.16 − 94.4)

Mean ± SD (range). *CL* centiloid, *MCI* mild cognitive impairment, *MMSE* minimental state examination, *SUVr* standard uptake value ratio

**Table 2:** Reproducibility of the visual and semi-quantitative assessments

All		VR			Equivocal		VR		
		P	N				P	N	
SUVr	P	39	9	48	SUVr	P	8	1	9
	N	3	75	78		N	2	10	12
κ = 0.7931		42	84	126	κ = 0.7123		10	11	21

All		VR			Equivocal		VR		
		P	N				P	N	
CL	P	39	5	44	CL	P	8	1	9
	N	3	79	82		N	2	10	12
κ = 0.8588		42	84	126	κ = 0.7123		10	11	21

*CL* centiloid, *N* negative, *P* positive, *SUVr* standard uptake value ratio, *VR* visual reading

## Discussion

In this study, a new technique was developed to quantify the accumulation of Aβ in the brain automatically using PET images alone, without anatomical imaging data such as MRI or CT. As the accumulation of Aβ in the brains of patients with Alzheimer's disease varies broadly, using a single template for anatomical standardization of PET images increases the error [25]. Edison et al. reported that the SUVr was overestimated if the quantification was performed with standardized PET images using an average template when compared to those performed with standardized MR images [26]. However, Bourgeat et al. reported that an optimal template, which is the weighted average image of the positive and negative templates to obtain the highest similarity to the participant PET image, can reduce the error of standardization for each patient [25]. We also found that the use of a single average template in the quantitative analysis of ^18^F-flutemetamol-PET images underestimated the values of SUVr in the high SUVr group and overestimated those in the low SUVr group and that the weighted average of the positive and negative PET images could improve these under/overestimations (Supplemental Fig. 1). However, we developed a new semi-quantitative analysis method to automatically calculate SUVr and CL values without any anatomical images and validated the accuracy of the spatial normalization of Aβ images. In the spatial normalization of PET images, we utilized a program package that includes a method to express (or represent) a deformation field using basis functions; this program was developed based on a previous report [26]. As a result, the composite SUVr calculated automatically with the current semi-quantitative analysis method showed a high correlation with that in CortexID Suite (R^2^ = 0.9657) and CL values (R^2^ = 0.9188) (Fig. 5a, b). Therefore, our method will enable the following:

- Semi-quantitative analysis of Aβ-PET images without anatomical data such as MRI or CT
- Easy calculation of SUVr and CL and evaluation of Aβ deposition on a uniform scale
- Reduction of inter-operator variability of data and improvement of reproducibility through automation of data processing

As shown in Figs. 5b and 6b, although there was a significant difference in the CL values between cases evaluated visually to be negative and equivocal, their ranges overlapped and it was difficult to discriminate the two groups by even quantitative analysis using CL values. In contrast, cases that were positive and both negative and/or equivocal in visual reading could be distinguished by CL values, and the sensitivity and specificity at the optimal cutoff CL value of 12.400 were 94.0% and 92.9%, respectively, if the evaluation of visual reading was used as SOT. In the ROC analysis for SUVr and CL values, both the sensitivity and specificity at the optimal cutoff values were higher in the CL values (Fig. 7), which may be due to the difference in the shape of the VOIs and standard brain used for calculating each semi-quantitative parameter. While CL VOIs were created with ^18^F-FDG-PET [13] and values were calculated directly from ^18^F-flutemetamol-PET images, which were deformed referring to the MNI standard brain (asymmetrically shaped structure), the SUVr values were calculated in two steps: deformation of the PET images with reference to the MNI standard brain, followed by transformation of the deformed PET images to fit the Talairach standard (symmetry) and VOIs were created. Since the shape of the human brain is asymmetrical, the process of deformation from the MNI to the Talairach standard brain probably affected the sensitivity and specificity calculated based on the SUVr. Therefore, we believe that if SUVr could be calculated using the same methodology as that used to calculate CL, the sensitivity and specificity of SUVr would be equivalent to those of CL. Furthermore, the VOIs used to measure CL were created by identifying regions specific for the positive group by comparing two groups, namely positive and negative Aβ deposition, whereas the VOIs for each brain region used to calculate SUVr were generated based on anatomical locations, referring to the Talairach Daemon. In other words, although the VOIs for each region excluded white matter, they did not exclusively consist of pixels specific to the Aβ-PET-positive group but also included some pixels in the VOIs that involved high or low accumulation in both the positive and negative groups. These differences in creating VOIs used in our study probably also affected the calculation of sensitivity and specificity at the optimal cutoff values for SUVr and CL.

Of the 21 cases assessed to be equivocal by visual reading, 12 and 9 cases were objectively classified as negative and positive, respectively, by the optimal cutoff values of both SUVr and CL. Furthermore, the reproducibility (Cohen's kappa = 0.7123) of the evaluation between visual reading and our semi-quantitative analyses for the equivocal cases was acceptable. These results suggest that the current semi-quantitative evaluation is useful as an assistant tool for the visual reading of ^18^F-flutemetamol-PET images (the global standard for the evaluation of ^18^F-flutemetamol-PET imaging is visual reading.).

There were three cases in which the CL values were below the optimal cutoff value (CL = 6.6, 3.8, and − 4.2) although the visual readings were positive. In the two cases with CL = 6.6 and 3.8, Aβ accumulation was visually observed only in the temporoparietal lobe and posterior cingulate gyrus in addition to a part of the composite VOI, respectively, which could not be assessed as positive for Aβ accumulation in the quantitative analysis. Moreover, because the visually positive cases with negative CL values showed a significant accumulation of ^18^F-flutemetamol visually in the occipital lobe, which is not included in the evaluation area of our composite VOI, there was a discrepancy between the visual reading and current semi-quantitative analysis method (Table 2).

The optimal cutoff value for CL in the current study was 12.400, which was lower than that reported by Amadoru et al. for participants with AD [27]. This may be because participants in our study were healthy participants and patients with MCI, in whom Aβ burden is at an early stage, supported by the report that the optimal cutoff for CL for healthy participants aged 70 and above was 11.9 [28]. Considering the importance of assessing Aβ deposition in the brain at an earlier stage in the treatment of AD, our semi-quantitative analysis technique of measuring Aβ in the brain using the CL scale is considered to be useful in the diagnosis of AD, and it is expected that our cutoff value will be useful in clinical use. However, it is important to conduct further studies to evaluate Aβ accumulation in various clinical stages of AD before the cutoff can be used for actual discrimination.

There are some limitations to our semi-quantitative evaluation of Aβ burden. As mentioned, participants with Aβ deposition in some cortical areas only (e.g., occipital lobe) that cannot be detected by our method are evaluated as false negatives. The software might be unable to exert anatomical standardization precisely in case of severe brain infarction or ventriculomegaly. Cases in which a part of the brain is out of the visual field of PET images would lead to a mistake in the standardization during data processing according to our methodology. Further, the protocol of Aβ-PET imaging must be fixed to achieve more accurate analytical results because SUVr and CL values may fluctuate if the time between the administration of ^18^F-flutemetamol and the initiation of imaging is not kept constant. In addition, data processing for anatomical transformation and semi-quantitative parameter estimation is complex and relatively time-consuming. Therefore, improving processing speed is a future challenge to satisfy the requirements for clinical practice.

In conclusion, our semi-quantitative analysis technique using ^18^F-flutemetamol-PET can be used to calculate SUVr and CL automatically without anatomical images. Moreover, it can also be used to interpret positive or negative Aβ burden in the brain objectively and homogeneously and serve as a supplemental tool for the visual reading of equivocal cases in routine clinical practice.

## Supplementary Information

Below is the link to the electronic supplementary material.

Supplementary file1 (DOCX 254 KB)
