# Supplementary material for: Automated semi-quantitative amyloid PET analysis technique without MR images for Alzheimer’s disease
Source: Ann Nucl Med. 2022 Jul 11;36(10):865–75. doi: 10.1007/s12149-022-01769-x (PMC9515054; doi:10.1007/s12149-022-01769-x)
Supplement: Supplementary file 1 — Supplementary file1 (DOCX 254 KB) [file 12149_2022_1769_MOESM1_ESM.docx]

**Supplemental data**

**Automated semi-quantitative amyloid PET analysis technique without MR images for Alzheimer’s disease**

Etsuko Imabayashi^1,2^, Naoyuki Tamamura^3^, Yuzuho Yamaguchi^3^, Yuto Kamitaka^1^, Muneyuki Sakata^1^, Kenji Ishii^1,*^

^1^ Research Team for Neuroimaging, Tokyo Metropolitan Geriatric Hospital and Institute of Gerontology, 35-2 Sakae-cho, Itabashi-ku, Tokyo, 173-0015, Japan.

^2^ Department of Molecular Imaging and Theranostics, Institute for Quantum Medical Science Quantum Life and Medical Science Directorate, National Institutes for Quantum Science and Technology (QST), 4-9-1 Anagawa, Inage, Chiba 263-8555, Japan.

^3^ Nihon Medi-Physics Co., Ltd., 3-4-10 Shinsuna, Koto-ku, Tokyo 136-0075, Japan.

**Supplemental Fig. 1** Comparison of SUVr in anatomical standardization of ^18^F-flutemetamol-PET images by using an average single template and using weighted average template.

Correlation between the anatomical standardization by deformation parameters using DARTEL and the anatomical standardization using in-house program in ^18^F-flutemetamol-PET images was examined as follows: The anatomic standardization parameters for the registered MR images, which was calculated by DARTEL in SPM8, were used for transforming the PET images aligned with MR images to deform the PET images into the standardized brain shape, followed by calculation of Composite SUVr. The resulting Composite SUVr through DARTEL and our semi-quantitative analysis method was plotted on the x-axis and the y-axis, respectively (for each PET image, SUVr was measured using Composite VOI). It is shown that the anatomical standardization with a single average template for all cases (yellow circle) tends to be an underestimation and overestimation of values in the high SUVr and low SUVr groups, respectively. On the other hand, the anatomical standardization using the optimal template for each subject adopted in our method (red circle) improved those tendencies: the coefficient of determination (R^2^) is closer to 1. These results support previous reports [1] [2], and furthermore, it shows the high accuracy of the anatomical standardization with our method.

DARTEL: Diffeomorphic Anatomical Registration Through Exponentiated Lie Algebra; SUVr: standardized uptake value ratio. VOI: Volume of interest

**REFERENCES**

1. Bourgeat P, Villemagne VL, Dore V, Brown B, Macaulay SL, Martins R, et al. Comparison of MR-less PiB SUVR quantification methods. *Neurobiol Aging.* 2015;36(Suppl 1):S159−66. doi: 10.1016/j.neurobiolaging.2014.04.033.

2. Edison P, Carter SF, Rinne JO, Gelosa G, Herholz K, Nordberg A, et al. Comparison of MRI based and PET template based approaches in the quantitative analysis of amyloid imaging with PIB-PET. *Neuroimage.* 2013;70:423−33. doi: 10.1016/j.neuroimage.2012.12.014.
